# Gamma oscillations and application of 40‐Hz audiovisual stimulation to improve brain function

**DOI:** 10.1002/brb3.2811

**Published:** 2022-11-14

**Authors:** Xixi Chen, Xiaolong Shi, Yuwei Wu, Zhiqing Zhou, Songmei Chen, Yan Han, Chunlei Shan

**Affiliations:** ^1^ Department of Rehabilitation Medicine, Yueyang Hospital of Integrated Traditional Chinese and Western Medicine Shanghai University of Traditional Chinese Medicine Shanghai China; ^2^ School of Rehabilitation Science Shanghai University of Traditional Chinese Medicine Shanghai China; ^3^ Engineering Research Center of Traditional Chinese Medicine Intelligent Rehabilitation Ministry of Education Shanghai China; ^4^ Department of Rehabilitation Medicine Shanghai No.3 Rehabilitation Hospital Shanghai China; ^5^ Department of Neurology, Yueyang Hospital of Integrated Traditional Chinese and Western Medicine Shanghai University of Traditional Chinese Medicine Shanghai China

**Keywords:** 40‐Hz, auditory stimulation, brain dysfunction, light stimulation

## Abstract

**Background:**

Audiovisual stimulation, such as auditory stimulation, light stimulation, and audiovisual combined stimulation, as a non‐invasive stimulation, which can induce gamma oscillation, has received increased attention in recent years, and it has been preliminarily applied in the clinical rehabilitation of brain dysfunctions, such as cognitive, language, motor, mood, and sleep dysfunctions. However, the exact mechanism underlying the therapeutic effect of 40‐Hz audiovisual stimulation remains unclear; the clinical applications of 40‐Hz audiovisual stimulation in brain dysfunctions rehabilitation still need further research.

**Objective:**

In order to provide new insights into brain dysfunction rehabilitation, this review begins with a discussion of the mechanism underlying 40‐Hz audiovisual stimulation, followed by a brief evaluation of its clinical application in the rehabilitation of brain dysfunctions.

**Results:**

Currently, 40‐Hz audiovisual stimulation was demonstrated to affect synaptic plasticity and modify the connection status of related brain networks in animal experiments and clinical trials. Although its promising efficacy has been shown in the treatment of cognitive, mood, and sleep impairment, research studies into its application in language and motor dysfunctions are still ongoing.

**Conclusions:**

Although 40‐Hz audiovisual stimulation seems to be effective in treating cognitive, mood, and sleep disorders, its role in language and motor dysfunctions has yet to be determined.

## Graphical abstract

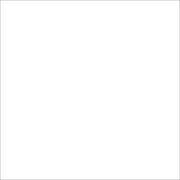

## 1 INTRODUCTION

Humans acquire information about the outside world primarily through vision and hearing, both of which account for 85% of all sensory afferents (Lukiw, 2020). The most essential and common audiovisual sensory inputs are exogenous sound and optical stimuli. The cerebral cortex reanalyzes and integrates sensory inputs in order to modulate brain function especially higher brain function.

Gamma oscillations are rhythmic activities generated by neurons, with frequencies ranging from 30 to 140 Hz, divided into three distinct gamma bands of slow (30–50 Hz), mid (50–90 Hz), and fast gammas (90–140 Hz) (Buzsáki & Wang, 2012). It has been shown that fast gamma is more active in exploring new things, whereas slow gamma prefers memory retrieval in familiar environments (Kay, 2003; Mably & Colgin, 2018) and dominates mid gamma to control recollection (Dvorak et al., 2018). Gamma oscillations are linked to functions, such as perception (Gurtubay et al., 2004), motor (Gross et al., 2005), attention, and memory (Gruber et al., 2004; Tallon‐Baudry et al., 2005). It has been demonstrated that gamma band modulated brain–muscle coherence most strongly especially in isometric contraction conditions when subjects were doing hands movement (Gross et al., 2005). Tallon‐Baudry et al. (2005) found that attention modulated gamma oscillations differently in different brain regions, for instance, gamma oscillations in fusiform gyrus increased under attentional stimulation, whereas gamma oscillations in the lateral occipital cortex enhanced during the preparation period before attentional stimulation. Gamma oscillations can be induced in the brain by sensory inputs, such as auditory stimulation, light stimulation, and audiovisual combined stimulation in the gamma band frequency. It has been demonstrated that acoustic and light stimulation at 40 Hz elicits a wider range of gamma neural oscillations than stimulation at other gamma band frequencies (Jones et al., 2019; Pastor et al., 2002) to modulate synaptic plasticity and neural networks and is currently being used in rehabilitation studies of various brain dysfunctions.

We conducted a search in PubMed and Google Scholar using combinations of medical subject headings and free words. The search terms included acoustic stimulation, photic stimulation, auditory stimulation, light stimulation, light flicker, visual stimulation, audiovisual stimulation, gamma oscillations, 40 Hz, cognitive, language, motor, mood, and sleep. The search included clinical trials and animal experiments as well as existing systematic reviews and meta‐analysis. There were no restrictions on publication date. The goal of this review is to provide a reference for future research on 40‐Hz audiovisual stimulation by briefly reviewing its mechanism of action and recent therapeutic applications in cognitive, language, motor, mood, and sleep disorders.

## 2 BASIC PRINCIPLES AND MECHANISMS OF AUDIOVISUAL STIMULATION

### 2.1 Auditory stimulation

Short or modulated sounds, which are repetitive, continuous, and fixed frequency auditory stimuli, can induce the same frequency of neural oscillations in the auditory cortex and thus improve relevant brain functions (Zaehle et al., 2010). Regular auditory stimulation has been shown to cause sustained high‐amplitude brain magnetic field changes in the auditory cortex, which promote synaptic gain and influence neuronal activity (Auksztulewicz et al., 2017). Additionally, 40‐Hz auditory stimulation was demonstrated to selectively activate the auditory areas of the pontocerebellum and increase regional cerebral blood flow to the contralateral auditory cortex, superior temporal gyrus (STG), and ipsilateral postcentral gyrus, as well as the inferior temporal gyrus (Pastor et al., 2002). There has been evidence that auditory stimulation at 40 Hz could cause gamma entrainment in the auditory cortex, hippocampus, and medial prefrontal cortex by modulating neuronal spiking activity. Furthermore, auditory stimulation resulted in related protein, glia, and blood vessel responses in Alzheimer's disease (AD) model mice, which had a crucial protective effect on neurons (Martorell et al., 2019).

### 2.2 Light stimulation

Flicker light with gamma band is typically utilized to visually stimulate participants in order to induce gamma oscillations. The power of the neural oscillations produced is influenced by the frequency, color, and intensity of the light stimulation. An animal experiment revealed that the visual cortex of cats generated steady‐state neural oscillations with light flicker stimulation ranging from 2 to 50 Hz, especially between 30 and 50 Hz, with significantly enhanced amplitudes (Rager & Singer, 1998). Herrmann (2001) observed that when flicker light stimulation frequency was set from 1 to 100 Hz in 1‐Hz steps, event‐related potentials presented steady‐state potentials with significant resonance phenomena around 10, 20, 40, and 80 Hz in 10 healthy volunteers. In the current state of clinical practice, studies have been mostly conducted with 40‐Hz light stimulation on cognitive function (Lin et al., 2021; You et al., 2020; Zhang et al., 2021). Specifically, light flickering at 34–38 Hz with high‐intensity red or white light has been shown to entrain stronger and wider gamma oscillations than lower intensity light stimulation (Jones et al., 2019; Lee et al., 2021; Noda et al., 2021).

In neurodegeneration model mice, Adaikkan et al. (2019) discovered that 40‐Hz light stimulation entrained gamma neural oscillations in the visual cortex (V1), hippocampal CA1, and prefrontal cortex, which modified synaptic plasticity‐related proteins, reduced neuronal and synaptic loss, and decreased neuroinflammation, thereby providing neuroprotection and delaying brain degeneration in mice. Forty‐hertz flicker leads to specific neuroimmune responses by upregulating the phosphorylation of proteins in the nuclear factor *κ*‐light‐chain‐enhancer of activated B cells and mitogen‐activated protein kinase pathways to increase an expression of cytokines (Garza et al., 2020). In addition, the 40‐Hz white light‐emitting diode boosted the activity of brain mitochondrial ATP‐sensitive potassium channel, as well as the structural and functional couplings of respiratory chain activity, enhancing mitochondrial function and neuroplasticity (Nazari et al., 2022).

Similar results for altered synaptic plasticity have been demonstrated in wild‐type mice (Tian et al., 2021). Moreover, 40‐Hz light flicker‐elicited gamma oscillations in hippocampal CA1 destabilized place cells by promoting the expression of long‐term depression; the destabilized place cells also improved spatial learning and memory performance in mice (Tian et al., 2021). In a cerebral ischemia murine model, the potential of 40‐Hz light stimulation to modulate synaptic plasticity was also demonstrated to have a neuroprotective effect (Zheng et al., 2020).

Some brain imaging studies demonstrate 40‐Hz light stimulation can functionally reorganize various brain regions and regulate the functional connectivity of related brain networks. An electroencephalography (EEG) study suggested that 40‐Hz violet optical stimulation induces an increase in the alpha–gamma coupling in the area F5 (left prefrontal area) during stimulation and in C4 (right central area) following treatment (Noda et al., 2021). Additionally, an EEG study showed that an increase was found in the connectivity between dorsolateral prefrontal and visual cortices under rhythmic visual stimulation (6‐, 10‐, 15‐, and 40‐Hz) compared with no increase could be found in no flicker condition (You et al., 2020). Forty‐hertz blue light stimulation has been shown to activate the visual cortex and hippocampus, improving the functional connectivity between the hippocampus and superior parietal lobe while decreasing the functional connectivity between the hippocampus and the default mode network (DMN), according to a functional magnetic resonance imaging (fMRI) study (Lin et al., 2021). Meanwhile, 40‐Hz light flicker could alter microstate metrics representing activation or deactivation states of brain networks with different functions and adjust functional state of brain networks to make them work (Zhang et al., 2021).

### 2.3 Audiovisual combined stimulation

It has been demonstrated that visual stimulation could affect the neural activity in the primary and secondary auditory cortices (Morrill & Hasenstaub, 2018), whereas auditory stimulation could also drive synaptic inhibition in the primary visual cortex through cortico–intercortical actions after activating the auditory cortex (Iurilli et al., 2012). The cross‐modality coordination between visual and auditory modalities can effectively characterize visual and auditory information perceptually (Mayer, 2003) and facilitate motor information processing (Soto‐Faraco et al., 2005). Compared with the information obtained via visual or auditory stimulation alone, information obtained via audiovisual integration can considerably shorten the time required for people to judge the target position (Nardini et al., 2016).

Audiovisual combined gamma flicker can downregulate tumor necrosis factor‐related weak inducer of apoptosis, transforming growth factor α, macrophage inflammatory protein 1β, Delta‐ and Notch‐like epidermal growth factor receptor, as well as immune factors such as interleukin‐5, in neural networks, thereby affecting neural activity and improving brain function of AD patients (He et al., 2021). Audiovisual combined stimulation induced more coordinated gamma oscillations that affect several brain regions more broadly and deeply than either visual or auditory stimulation alone (Suk et al., 2020). An animal experiment showed that amyloid beta plaques were generally reduced throughout the cerebral cortex under audiovisual combined stimulation at 40 Hz (Martorell et al., 2019). According to the findings of an EEG study, 40‐Hz auditory plus visual stimulation significantly increased the power spectral density of frontal and occipital neuron oscillations, was able to entrain gamma oscillations in deeper brain regions, such as the gyrus rectus, amygdala, hippocampus, and insula (Chan et al., 2021), and increased the phase theta–gamma phase‐amplitude coupling (Fatemi et al., 2022). According to fMRI studies, 40‐Hz synchronized auditory and visual stimulation delayed brain atrophy and strengthened functional connectivity between the whole brain and the DMN and medial visual network (MVN) (Chan et al., 2021), as well as between the posterior cingulate cortex and the precuneus (He et al., 2021).

In summary, microscopically, audiovisual stimulation in the gamma band alters synaptic plasticity and induces neuroimmune responses to provide neuroprotection. Macroscopically, it can modulate the functional status of brain regions, such as frontal, parietal, occipital lobes, and hippocampus in current evidence, and reconstruct the balance between brain networks, which can improve the efficiency of brain networks. These evidences all lay the foundation for the possibility of 40‐Hz audiovisual stimulation to ameliorate brain dysfunctions.

## 3 APPLICATION OF 40‐HZ AUDIOVISUAL STIMULATION IN THE REHABILITATION OF BRAIN DYSFUNCTIONS

### 3.1 Cognitive disorders

Studies on cognitive dysfunction with 40‐Hz audiovisual stimulation are still primarily at the experimental animal stage, with relevant clinical trial studies still in the initial stage. Gamma oscillations are involved in memory encoding and recognition. It has been shown that learning encoded items results in increased gamma band responses during subsequent memory recognition (Gruber et al., 2004). Moreover, stronger gamma oscillations are induced when a memory task stimulus is matched to an image that has already been remembered (Herrmann et al., 2004). Forty‐hertz audiovisual stimulation has been shown to improve learning and memory performance by modulating synaptic plasticity, inducing microglia responses, reducing neuroinflammation, providing neuroprotection in relevant model mice, inducing gamma oscillations, increasing the power of spontaneous gamma neural oscillations, and generally reducing beta amyloid plaques (Adaikkan et al., 2019; Lee et al., 2018; Martorell et al., 2019). When the auditory stimulation is both presented to each ear, they are called monaural beats (Engelbregt et al., 2021). In addition, they are called binaural beats when the one tone is presented to one ear and the other tone separately to the other ear. Binaural beat stimulation could induce an additional phantom frequency to be interpreted by the cerebral cortex by exposing a subject to two different, coherent tones operating at different frequencies. In addition, the frequency of the occurring beat is equal to the value of the difference between the frequencies of the tones received by the left and right ears (Kuwada et al., 1979; Schwarz & Taylor, 2005). In contrast to monaural beats, when perceiving binaural beats, the firing rate of oscillating brain activity is synchronized (Grose & Mamo, 2012). Sharpe et al. (2020) discovered that after 4 weeks of 25, 40, or 100 Hz binaural beat stimulation in three groups of healthy subjects, the mean improvement of memory scores in the 40‐Hz group were statistically significant, elevating from an average of 87%–95%. Similar result was also found in Jirakittayakorn's study, which showed that after 40‐Hz binaural beat stimulation, the recalled words were increased in the working memory part of the list (Jirakittayakorn & Wongsawat, 2017). In addition, experimental results in healthy subjects suggest that rhythmic visual stimulation can facilitate attention. The study shows compared with no background flicker, jittered flicker, 6‐, 10‐, and 15‐Hz rhythmic visual stimuli, volunteers receiving 40‐Hz rhythmic visual stimuli achieved the best performance and the fastest reaction time in attention visual tasks in healthy subjects (You et al., 2020).

Short‐term audiovisual stimulation may not have a meaningful effect in patients with neurodegeneration, but long‐term audiovisual stimulation is safe and feasible. Amyloid β precipitation and tau aggregation are linked to AD (Avila, 2006; Jakob‐Roetne & Jacobsen, 2009). Compared to 4 weeks of treatment, after 8 weeks of 40‐Hz audiovisual combined stimulation in a feasibility study, substantial changes in immune factor levels in the cerebrospinal fluid and brain functional networks were detected in AD patients (He et al., 2021). A randomized controlled trial found increased functional connectivity between the DMN and MVN, as well as between them and the whole brain after 3 months of combined 40‐Hz acoustic and light stimulation in patients with probable mild AD, with improved performance on the face‐name association delayed recall test (Chan et al., 2021). However, it has also been demonstrated that the levels of amyloid beta plaques and tau are not reduced after a period of light or audiovisual combined stimulation of AD patients (He et al., 2021; Ismail et al., 2018).

In conclusion, the above studies, mainly in AD patients, have preliminarily demonstrated that 40‐Hz audiovisual stimulation promotes attention, learning, and memory. However, its effect on patients with cognitive dysfunction is influenced by the treatment course, which indicates 40‐Hz audiovisual stimulation has a cumulative effect. Further studies on its action duration, as well as its long‐term and short‐term efficacies, need to be performed in subsequent large‐sample trials in the future.

### 3.2 Language disorders

Forty‐hertz audiovisual stimulation could affect language function, and its probable mechanism of action should be explained from the relevant language processing, specific brain regions, and the specific frequency oscillations.

Language function is mainly divided into two parts: speech perception and speech output. Speech perception is the process of extracting auditory and visual information, mainly related to the STG and posterior superior temporal sulcus (pSTS). A study has shown that the 65–100 Hz gamma oscillations power in STG was connected to the increase in auditory stimulation power, with the increase being more substantial under acousto‐optic conditions. Compared with the single‐peak auditory speech perception, there are at least three different activity modes in the STG during the audiovisual speech perception. In these three modes, the gamma power will continuously increase at posterior STG before the sound starts to reach the peak. However, because visual information of speech occurs before auditory information, the increase in gamma power occurs only before speech initiation and exclusively in the posterior STG (Karthik et al., 2021). Marchesotti et al. (2020) found that phonological deficit in dyslexia patients was related to the change of low‐gamma oscillation function of left auditory cortex, leading to no causal relationship between the oscillation function and phoneme processing. They used a 30‐Hz low‐gamma frequency transcranial alternating current stimulation (tACS) to intervene the patients and observed that the patients’ phonological processing and reading accuracy had been significantly improved. It can be seen that the low‐gamma frequency intervention has a corresponding effect on the perception and processing of speech.

Studies on neural substrates of visual signals transforming to an auditory system mainly focus on left pSTS. pSTS distinguishes between pure auditory stimulation and audiovisual combined stimulation and plays a potential causal role in audiovisual speech integration (Ozker et al., 2018). Alho et al. (2014) used magnetoencephalography to confirm that the synchronization of gamma oscillations across brain regions is stronger in active listening (initiate speech processing with paying attention to the speech) than in passive listening (without paying attention to the speech). These findings revealed the importance of the audiovisual speech perception model, as well as the significance of audiovisual combined stimulation with gamma band, for speech perception module.

Speech output includes repeating, naming, and expression. Current research on speech output is more detailed and in‐depth, observing not only an individual's ability of repeating and naming but also their language‐processing ability in terms of semantics, grammar, and pragmatics. Gamma oscillations have been found to represent the degree of lexical semantic synchronization generated by beta oscillations in context prediction (Lewis & Bastiaansen, 2015; Lewis et al., 2016) while also involving in checking the accuracy of vocabulary used in sentences and the completeness and completion of sentences. In other words, gamma oscillations cannot be synchronized if the semantics of incoming words do not match the semantics predicted by the previous context (Meyer, 2018). Wang et al. (2018) compared the neural oscillations generated by the “expectancy” of words appearing in sentences and discovered that inconsistent words could induce an increase in the power of gamma oscillations in the left frontal and temporal lobes. This result is somewhat different from previous studies and may be related to the composition of the stimuli set in a particular experimental setting, such as the prediction comparison of high constraint and low constraint contexts. Through these studies, we can find that the majority of gamma oscillations studies are related to lexical retrieval, syntactic processing, and context integration, which tend to focus on semantics and pragmatics—that is, more complex and advanced speech abilities.

There is currently no clinical study regarding 40‐Hz audiovisual stimulation on language impairment. However, according to a study of speech‐related gamma oscillations, we found that gamma oscillations are substantially linked with an individual's speech perception and semantic syntax processing, which happens to be one of the more difficult problems in clinical language disorders. Based on the fact that audiovisual combined stimulation has been linked to gamma oscillations of STG, 40‐Hz audiovisual stimulation may have certain effects on patients with auditory comprehension dysfunction (such as sensory aphasia and pure word deafness). The frontal, occipital, and temporal lobes have been demonstrated to be affected by 40‐Hz audiovisual stimulation in studies (Lee et al., 2018; Pastor et al., 2002), which are important brain areas related to speech and language. Therefore, we can speculate that 40‐Hz audiovisual stimulation may have an impact on pragmatic‐related functions, such as lexical retrieval and syntactic processing, but further research is needed.

### 3.3 Motor disorders

Beta and gamma oscillations are mainly present in the motor cortex. Soteropoulos and Baker (2006) found that the cerebellum and motor cortex generate synchronous neural oscillations. When controlling finger movement and preparing for finger movement, healthy adults will generate 40‐Hz neural oscillations in the motor cortex (Salenius et al., 1996). The neural oscillations in the motor cortex are shifted toward the gamma‐range during isotonic contractions of the lower limb muscles and then are shifted toward the beta‐range (13–30 Hz) (Gwin & Ferris, 2012). Previous studies have demonstrated that tACS delivered at the gamma frequency can modulate the long‐term potentiation‐like plasticity of the primary motor cortex, proving that driving cortical gamma oscillations can improve motor performance (Guerra et al., 2019, 2020; Nowak et al., 2017). A study showed that gamma‐oscillation (70 Hz) tACS were more effective in improving motor performance compared to beta‐oscillation (20 Hz) tACS (Miyaguchi et al., 2018). However, there are no studies on 40‐Hz audiovisual stimulation for motor function rehabilitation. Although the findings described below are not about 40‐Hz stimulation, the resulting auditory‐motor entrainment and the driving effect on the motor system lay the foundation for exploring whether 40‐Hz audiovisual stimulation is a better choice. Existing studies on motor function rehabilitation using audiovisual stimulation are still in the early phases. Malcolm et al. (2009) found that rhythmic auditory stimulation (auditory cues given by a 1000‐Hz digital metronome) for stroke patients could improve their upper limb motor function. In an EEG study, 40 young healthy subjects were divided into an auditory‐only condition, motor‐only condition, and rhythmic auditory‐motor synchronization condition (1.25 Hz) (Crasta et al., 2018). This experiment showed that neural oscillations generated from auditory stimulation (under auditory‐only condition) led to the appearance of auditory‐motor entrainment and thus drove the motor system. Furthermore, under an auditory‐motor synchronization condition, subjects exhibited higher neural activity efficiency under the auditory‐induced condition compared with motor‐induced condition.

Schoffelen et al. (2005) proposed that gamma oscillations at 40–70 Hz were involved with the interaction between vision and motor function, whereas Franz et al. (2017) found that visuomotor entrainment (movement generated in synchrony with visual stimuli in human feedback) is closely related to the walking process. It involves the coordination and frequency adjustment of trunk and lower limb movements, which aids in maintaining balance. An animal experiment revealed that in monkeys, the supplementary motor area was involved in generating motor plans that dynamically match rhythmic visual stimulation (Ivry, 2018). The ventral pathway of visual perception can help to recognize objects, whereas the dorsal pathway can help to analyze the spatial structure of objects and identify their location. The two pathways work together to guide actions (Michael & Gazzaniga, 2009). Visual stimulation at 32–50 Hz has been shown to induce gamma oscillations in the parietal and frontal lobes (Park et al., 2022), and to activate temporal lobes because of cross‐sensory effects (Raij et al., 2010), impacting both dorsal and ventral pathways to improve motor behavior.

Taken together, we believe that audiovisual stimulation, especially with specific frequency, has a certain impact on the rehabilitation of motor dysfunction. Visuomotor entrainment or auditory‐motor entrainment, either induced by visual or auditory stimulation, may drive the motor system to produce a top–down control, whereas motor training produces a bottom–up feedback to activate motor system. The combination of audiovisual stimulation and exercise therapy, such as audiovisual stimulation preceded by exercise therapy or simultaneous audiovisual stimulation and exercise therapy, may be a more beneficial treatment for improving motor function compared to exercise therapy alone (Lee et al., 2015; Pan et al., 2018). Because the combination of the two may produce top–down and bottom–up dual feedback, which produces higher neural activity efficiency. To fill the gap in clinical studies of 40‐Hz audiovisual stimulation on motor function, future studies need to pay more attention to clinical application of 40‐Hz audiovisual stimulation combined with exercise therapy in motor function rehabilitation, in order to explore the effect of audiovisual stimulation combined with the timing and intensity of adding exercise therapy on patients, as well as the mechanism of up‐ and downregulation of audiovisual stimulation combined with exercise therapy.

### 3.4 Mood and sleep disorders

Compared to the other frequency bands, 40‐Hz audiovisual stimulation could also be effective for mood (Sharpe et al., 2020). A study showed that AD patients receiving 40‐Hz audiovisual stimulation exhibited reduced anxiety/agitation compared with patients who received digital video disc (DVD) treatment (Clements‐Cortes et al., 2016). The DVD treatment was designed to calm AD patients, but it was not as effective as 40‐Hz audiovisual stimulation. Jirakittayakorn and Wongsawat (2017) used Brunel Mood Scale to confirm the effect of 40‐Hz auditory stimulation on mood, and they found that stimulation duration decides mood states. Duration that is too long leads to negative effects, such as fatigue. Although a suitable duration (in this experiment, 20 min is better than 30 min) leads to positive effects, such as decreases of tension and depression and increase of vigor. Future research should pay special attention to the impact of the duration of audiovisual stimulation on patients, explore the optimal stimulation duration, avoid causing discomfort to patients, and thus affecting the therapeutic effect.

Chan et al. (2021) proposed that 40‐Hz audiovisual stimulation improved sleep quality for AD patients by increasing normal sleep time and reducing fragmented sleep time. Another study also showed that 40‐Hz audiovisual stimulation improved sleep quality for AD patients, whereas the sham group showed a decrease in sleep quality (Cimenser et al., 2021). An EEG study suggested that during alert wakefulness, the interhemispheric coherence in the low gamma (30–45 Hz) frequency band is greater than that of quiet wakefulness (Castro et al., 2014). In addition, when it comes to rapid eye movement sleep (which is a deeper state of sleep), there is almost no EEG coherence in the gamma band spectrum (30–100 Hz) (Castro et al., 2013; Torterolo et al., 2016). The above findings suggest that the 40‐Hz audiovisual stimulation may somehow induce the gamma coherence in the brain and is helpful for improving sleep quality, but further EEG studies are needed to investigate the detailed principles.

All 40‐Hz audiovisual stimulation clinical studies mentioned before are summarized in Table 1.

**TABLE 1:** Forty‐hertz audiovisual stimulation clinical studies in brain dysfunction rehabilitation

References	Sample size (*N*)	Domain	Group	Cohort	Stimulation	Duration and course	Outcomes
Chan et al. (2021) (experiment1)	41	Cognitive; sleep	None	Cognitively normal adults, adults with a previous diagnosis of probable AD, and neurosurgical patients with medically intractable epilepsy	Audiovisual combined stimulation	3–9 min, not mentioned	EEG
Chan et al. (2021) (experiment2)	15	Cognitive; sleep	Active group and placebo group	Patients with mild probable AD dementia	Audiovisual combined stimulation	1 h, 9 months	Neuropsychiatric testing, EEG, MRI, and blood collection for sequencing
Ismail et al. (2018)	6	Cognitive	None	AD patients and one MCI patient	Light stimulation	1 h in morning, 1 h in evening, 10 days	PET, MRI
You et al. (2020)	33	Cognitive	None	Healthy participants	Light stimulation	Not mentioned	EEG, RT
He et al. (2021)	10	Cognitive	4‐week group and 8‐week group	Participants with MCI due to AD	Light, auditory and audiovisual combined stimulation	1 h, 4 or 8 weeks	Venous blood draws, lumbar punctures, MRI, EEG, and cognitive testing
Jirakittayakorn and Wongsawat (2017) (experiment1)	7	Mood	None	Healthy participants	Auditory stimulation	30 min, not mentioned	EEG, Brunel Mood Scale
Jirakittayakorn and Wongsawat (2017) (experiment2)	40	Cognitive	None	Healthy participants	Auditory stimulation	20 min, not mentioned	Word list recall task
Cimenser et al. (2021)	22	Sleep	Active treatment group and sham group	Patients with mild‐to‐moderate AD	Audiovisual combined stimulation	1 h, 6 months	Restful and active periods during sleep, ADCS‐ADL scale
Clements‐Cortes et al. (2016)	18	Mood	None	AD patients	Auditory stimulation	30 min, 6 sessions	SLUMS, observed emotion rating scale, and researcher observation

Abbreviations: AD, Alzheimer's disease; EEG, electroencephalography; MCI, mild cognitive impairment; MRI, magnetic resonance imaging; PET, positron emission tomography; RT, reaction time; SLUMS, Saint Louis University Mental Status.

## 4 SAFETY AND PRECAUTIONS

Most clinical trials for 40‐Hz audiovisual stimulation are feasibility studies, and safety issues still require more investigation. White light and brief auditory clicks or tones of 67–80 dB are commonly used in 40‐Hz audiovisual stimulation treatments (Adaikkan & Tsai, 2020); some studies have suggested that although high‐intensity flickering light can induce gamma oscillations with a wider spectrum, it is also more likely to cause adverse effects, such as fatigue, dizziness, and eye pain. As a result, 400 cd/m^2^ of moderate intensity flicker light is indicated for intervention (Lee et al., 2021). In order to minimize the foregoing negative impacts, several studies have developed an alternative approach with 40‐Hz invisible flicker (Agger et al., 2022). Although invisible light stimulation can potentially lead to gamma oscillations in brain regions, more thorough research is still required to confirm any possible placebo effects.

For audiovisual combined stimulation, light and sound should be presented simultaneously at the same onset time (Chan et al., 2021; Martorell et al., 2019). Nevertheless, a study demonstrated that the subjects could not consciously perceive the subtle difference in phase synchronization between auditory and visual stimuli (±125 ms). Considering that auditory responses are a few milliseconds faster than visual responses, researchers believe that auditory stimulation should be delayed when audiovisual combined stimuli are applied to people (Clouter et al., 2017). When visual and auditory stimulations are delivered with delays ranging from 50 to 300 ms, the behavior of healthy subjects discriminating between synchronized and delayed audiovisual integration stimulation led to the activation of a large‐scale neural network consisting of insula, posterior parietal, prefrontal, and cerebellar regions (Bushara et al., 2001). It implies that when individuals perceive a slight delay in audiovisual combined stimulation, the resulting neural network activation may have an impact on the intervention effect.

To address the demands of clinical applications, more studies on audiovisual combined stimulation parameters are required. Existing studies have shown that 40‐Hz audiovisual stimulation improves cognitive performance in patients with neurodegeneration such as AD. Additionally, 40‐Hz light stimulation has been demonstrated to improve the cognitive performance of mice with cerebral ischemia, which opens up the possibility of clinical trials. Although there are limited studies regarding the application of 40‐Hz light or/and auditory stimulation in the rehabilitation of patients with brain injury, such as stroke, the risk of epilepsy needs to be taken into account when conducting preliminary exploratory experiments (Hughes, 2008).

## 5 CONCLUSION AND FURTHER PROSPECTS

Gamma oscillations have been shown to be closely related to cognitive function. Audiovisual stimulation at 40 Hz induces gamma oscillations and enhances the synchronization of gamma oscillations, which has the effect of improving cognitive function. Audiovisual stimulation has unique advantages in the field of brain disease rehabilitation due to its non‐invasive nature. Clinical studies on 40‐Hz audiovisual stimulation are limited and are mostly associated with cognitive rehabilitation and gradually show the potential for improving mood and sleep. However, 40‐Hz audiovisual stimulation can affect the frontal, occipital, parietal, and temporal lobes, indicating its potential value in the rehabilitation of other functions, such as language and motor. Although its specific mechanism of action is still unclear, the light color, light intensity, and timing or duration of delivery during audiovisual combined stimulation need to be further determined. Therefore, more high‐quality, large‐sample studies need to be conducted in the future to explore the mechanism and clinical efficacy of 40‐Hz audiovisual stimulation for the rehabilitation of brain dysfunction.

## AUTHOR CONTRIBUTIONS

Xixi Chen and Xiaolong Shi conceptualized and wrote original draft; Yuwei Wu, Zhiqing Zhou, and Songmei Chen were involved in literature research and drafting; Chunlei Shan and Yan Han designed and supervised the manuscript; all authors reviewed the manuscript and provided critical revision.

## CONFLICT OF INTEREST

The authors have no conflicts of interest to disclose.
